# Effects of Non-Thermal Plasma Treatment on Plant Physiological and Biochemical Processes, Second Edition

**DOI:** 10.3390/plants15040546

**Published:** 2026-02-10

**Authors:** Bozena Sera, Vida Mildaziene

**Affiliations:** 1 Department of Environmental Ecology and Landscape Management, Faculty of Natural Sciences, Comenius University Bratislava, 84215 Bratislava, Slovakia; 2 Faculty of Natural Sciences, Vytautas Magnus University, LT-53361 Kaunas, Lithuania; vida.mildaziene@vdu.lt

Research on plasma interactions with seeds is driven by the growing global demand for food and agricultural products under conditions of limited resources [1]. Developing environmentally friendly technologies to enhance crop production is essential for reducing unsustainable use of water, nutrients, and agrochemicals [2]. Pre-sowing seed treatments have long been employed to improve germination and seedling growth. Recently, non-thermal atmospheric pressure plasma (NTP) technologies have attracted increasing attention due to their reported positive effects on plant performance [3,4].

NTP sources were first developed over three decades ago [5] and have since been applied across various industries to modify the physicochemical properties of solids, liquids, and microparticles [6]. A fundamental property of plasma is its ability to generate reactive species that initiate physical and chemical processes otherwise difficult to achieve [7]. Owing to these properties, NTP has been widely explored for sterilization, water purification, microfabrication, medicine, seed technology, and agriculture [8,9]. In the agricultural context, NTP has shown potential to improve seed quality through surface decontamination, enhanced seed germination, and plant’s growth stimulation [10,11,12,13,14]. Plasma-activated water (PAW), another promising application, is gaining attention for its antimicrobial properties and potential use in agriculture, food safety, and sterilization [15].

However, despite numerous laboratory and small-scale field studies, the underlying molecular mechanisms remain poorly understood, and large-scale applications require further development. This Special Issue “Effects of Non-Thermal Plasma Treatment on Plant Physiological and Biochemical Processes, Second Edition” is a continuation of the previous successful issue of the journal Plants [4] and was created with the aim of contributing to the understanding of the impact of both NTP and PAW on plants, at all levels of existing multidimensional research. This Special Issue contains six original experimental studies (four papers on NTP, two papers on PAW) and one critical review, which will be now briefly introduced.

NTP and plant papers

Scholtz et al. [16] demonstrated that black locust (*Robinia pseudoacacia*) seeds exhibit strong physical dormancy caused by a waxy seed coat. Treatments using various NTP sources [including corona discharge, back corona discharge, argon atmospheric-pressure plasma jet, and dielectric barrier discharge (DBD)] significantly increased surface hydrophilicity, reduced water contact angle from 120° to complete wetting, and induced germination, whereas untreated seeds failed to germinate. The most effective method was DBD, which completely removed the wax layer and achieved over 80% germination. Surface alterations were attributed to oxidation and hydrophilization of the wax layer and the formation of channels facilitating water penetration.

Similar trends were observed in other species. Tunklová et al. [17] investigated the effects of NTP on durum wheat (*Triticum durum*) grains and common buckwheat (*Fagopyrum esculentum*) achenes using diffuse coplanar surface barrier discharge (DCSBD) and microwave surface barrier discharge (MSDBD) for 3–40 s. Surface diagnostics revealed a significant reduction in water contact angle, indicating improved wettability, while attenuated total reflectance (Fourier transform infrared spectroscopy analysis) confirmed negligible changes in chemical composition, supporting the non-destructive nature of NTP. Multivariate analysis of twenty germination and early growth parameters showed positive, though statistically non-significant, trends. Optimal exposure times differed between species: common buckwheat responded best to MSDBD for 5–10 s, whereas durum wheat required 10–20 s. The root-to-shoot dry matter ratio emerged as a key trait, suggesting enhanced root investment, which may improve drought resilience.

Motrescu et al. [18] highlighted species-specific responses of alfalfa (*Medicago sativa*) to NTP. Using a flexible electrode configuration suitable for in-package processing, significant microstructural changes were observed on seed surfaces. Biometric measurements indicated that certain treatment conditions promoted sprout growth by up to 50% compared to controls; however, biochemical analyses revealed variable responses in chlorophyll, flavonoid, polyphenol content, and antioxidant activity. Most treatments exhibited inhibitory effects, suggesting that NTP acts as a stress factor and that optimal conditions or milder, indirect treatments are essential for consistent stimulation.

Beyond germination and growth, NTP may also influence secondary metabolite biosynthesis. Judickaite et al. [19] demonstrated that pre-sowing plasma treatment of stevia (*Stevia rebaudiana*) seeds significantly enhanced steviol glycoside (SG) accumulation, particularly after 5 min exposure, which resulted in a 2.5-fold increase in rebaudioside A and a 1.6-fold increase in stevioside. Total phenolic content, total flavonoid content, and antioxidant activity remained unchanged. NTP treatment tended to reduce plant height and leaf dry mass, while correlation analysis revealed negative associations between certain morphometric traits and SG concentrations, suggesting that morphological parameters may partially predict plasma-induced biochemical responses.

PAW and plant papers 

Ferreyra et al. [20] evaluated the effects of PAW on tomato (*Solanum lycopersicum*) and bell pepper (*Capsicum annuum*) under greenhouse conditions. PAW was generated using a glow-type discharge plasma reactor with a water cathode, achieving high concentrations of reactive oxygen and nitrogen species after 15 min of activation: 20 mg/L H_2_O_2_, 147.6 mg/L NO_2_^−^, and 182.3 mg/L NO_3_^−^, with energy efficiencies among the highest reported (60 nmol/J for NO_x_ synthesis). PAW irrigation significantly enhanced bell pepper seed germination by up to 26%, whereas tomato seeds showed no improvement. Growth promotion was observed in both crops, with dry weight increases of 44–61% in bell pepper and 11–39% in tomato. Importantly, no oxidative damage was detected, and antioxidant enzyme activity indicated adequate defence responses, confirming PAW as a safe and eco-friendly alternative for improving crop performance without chemical additives.

In orchard crops, Kuzin et al. [21] investigated PAW effects on apple (*Malus domestica*) scion survival, vegetative growth, and mineral nutrition under field conditions. Two PAW treatments (PAW1 and PAW2) at concentrations of 50 and 100 mL/L were applied during the pre-harvest period. PAW slightly improved fruit yield by reducing fruitlet loss, particularly in PAW2 (100 mL/L) and PAW1 (50 mL/L) treatments. Foliar nitrogen content increased at lower PAW concentrations, while higher doses tended to inhibit nitrogen metabolism. PAW2 had the most pronounced effect on leaf N, K, and Ca, and fruit Ca content increased significantly after PAW1 (100 mL/L) and PAW2 (50 mL/L), potentially enhancing storability. However, no significant improvement was observed in graft survival or annual shoot elongation, with rootstock thickness remaining the dominant factor. Overall, PAW demonstrated modest positive effects on yield and nutrient uptake, warranting multi-year studies to clarify its long-term impact on apple tree performance.

Review 

Doshi et al. [22] critically reviewed the current state of research on NTP applications for fungal control and toxin decontamination. Literature analysis indicates that NTP effectively reduces *Fusarium* sp. viability and degrades mycotoxins, with treatment efficiency strongly influenced by plasma parameters such as gas composition, voltage, power, and exposure time. Proposed mechanisms include oxidative damage to fungal cell structures and chemical modification of mycotoxin molecules through reactive species generated during plasma discharge.

Conclusion 

Plasma-based technologies, including non-thermal plasma (NTP) and plasma-activated water (PAW), offer significant potential for advancing sustainable agriculture. NTP has demonstrated effectiveness in overcoming physical seed dormancy, enhancing germination, and, in some cases, modulating metabolic pathways. Similarly, PAW has shown promise as an eco-friendly alternative for improving crop productivity and nutritional quality, aligning with resource-efficient production goals. Both technologies, however, exhibit species-specific responses and strong dependence on treatment parameters, underscoring the need for optimization and mechanistic understanding.

Beyond plant growth stimulation, NTP also holds potential for pathogen control and mycotoxin decontamination, providing a physical, chemical-free strategy that could complement biological approaches within integrated pest management frameworks. Despite encouraging results, these technologies remain at an early stage of development. Future research should focus on scaling up applications, refining treatment protocols, and assessing long-term impacts under field conditions to ensure efficacy, safety, and economic viability.

## Data Availability

All the data included in the main text.
